# Laryngology and Rhinology

**Published:** 1892-06

**Authors:** Barclay J. Baron


### LARYNGOLOGY AND RHINOLOGY.

To arrive at an accurate diagnosis of disease of the antrum is sometimes a difficult matter. The method of exploration and irrigation of this cavity suggested by Lichtwitz[4] promises to be of value in this connection. The instrument to be used consists of a straight trochar, having a diameter of one millimètre and a half with a length of ten to twelve centimètres, in a steel cannula. After the nostril has been cleansed by means of an antiseptic douche, and the

[4] *Journ. Laryn. & Rhin.*, Dec., 1891., p. 485.

inferior meatus cocainised, puncture of the antrum is made in the following manner: the instrument is passed into the nose as far as the middle of the inferior meatus, and the point, which is directed as much outwards and upwards as possible, is then pushed through the thin inner wall of the antrum. Syringing through the cannula drives out the fluid contents of the antrum through the normal opening, and they can thus be examined by sight and smell.

As we all know, pus in the antrum is usually produced by a carious condition of a molar tooth, and Luc [1] has asserted that dental suppuration *always* starts the abscess. He has, however, published two cases,[2] in one of which no injury to the molar teeth could be discovered until extraction of the first one revealed old alveolar dental periostitis; and in the other case there was merely a small superficial cavity in the crown of the first molar, but the same condition of the fangs was seen on extraction. Whenever a patient consults us for discharge of pus from the nose, we ought to first inquire, Is it unilateral or bilateral? If the former, and if coming from under the middle turbinated bone, it is pretty certainly of antral origin. We shall usually find that there has been toothache, and we often see a decayed or dead first molar, but absence of the dental signs does not negative the diagnosis of antral mischief.

Dr. Middlemass Hunt [3] has published two cases, showing how very important is accurate examination of the interior of the nose. They were cases of common cold, spreading to the accessory sinuses, and giving rise to the characteristic symptoms, which were very severe as regards pain. There was discharge of foul-smelling pus from one nostril, and on examination this was found to be packed with stinking cheesy pus, which was prevented from making its exit by a hypertrophied fold of mucous membrane springing from the external wall, just in front of the middle turbinate. Antiseptic irrigation, scooping out the pus, and application of the galvano-cautery to the hypertrophied valve-like flap cured what was looked on as a most serious condition.

I have dealt with one or two cases of a similar kind, and find that it is absolutely necessary that the discharge be removed by the medical attendant until it is *easily* got rid of by the nasal syringe. I insist on my patients getting an instrument the nozzle of which is pierced with five holes, so

[1] *Arch. de Laryn.*, 1890, pp. 145–204.
[2] See *Journ. Laryn., Rhin., & Otol.*, Jan., 1892, p. 38. [3] *Ibid.*, p. 1.

that the stream of water radiates in a fan-like manner, and cleanses the whole cavity thoroughly. The galvano-cautery, chromic acid, or the snare must be used to get rid of the flap that is causing the retention of foul secretions.

At a meeting of the Society of Laryngology and Otology of Paris, held on November 6th, 1891, Dr. Gellé [1] reported a case in which suppurative otitis and cerebral symptoms supervened after posterior tamponing for severe epistaxis. The ear and brain mischief was caused by the retention of putrid clot in the tampon, and it is a thoroughly practical and important point to remember that this may take place. It is in reality very rarely necessary to plug the posterior nares for epistaxis, as the blood almost always issues from the anterior part of the septum; and I have seldom failed to find the bleeding point, and plug it effectually by means of a bead of chromic acid melted on the end of a nasal probe, and applied to the spot whence the blood is issuing. Dusting the septum over with iodoform, or the application of a plug of iodoform cotton wool, promotes healing of the little wound, and is the only scientific and safe way of treating this trouble.

At a meeting of the same Society, on December 4th, 1891, Dr. Albert Ruault [2] read a paper on "Superficial Submucous Phlegmon of the Base of the Tongue." The symptoms of the affection are malaise, headache, shivering, and fever lasting four to seven days, pain in the throat, shooting into the ears; in some cases, hoarseness and dyspnœa. There is no swelling of the neck and no glandular enlargement. The patient can open the mouth widely, and the faucial tonsils not being swollen, we must examine with the laryngoscope. The appearances of the lingual tonsil are those of the faucial tonsil when inflamed; viz., redness, swelling, and, if suppuration has supervened, spots of pus on its surface, with, in most cases, œdema of the epiglottis. The abscess opens only after several days, or, if there be merely non-suppurative inflammation, it gradually recedes. The treatment consists in scarification or deeper incision, antiseptic gargles, and poultices.

I have had two such cases under my care, in one of which the lingual tonsil was as large as a hazel-nut, red and shiny in appearance, and intensely painful, but it could only be seen with the laryngoscopic mirror, and it gradually receded;

[1] See *Journ. Laryn., Rhin., & Otol.*, Jan., 1892, p. 40.
[2] *Ibid.*, March, 1892, p. 135.

in the other it opened laterally, and discharged a large quantity of pus. When we get a patient suffering from pain in the throat, the origin of which we cannot see on looking into the mouth, we ought on no account to omit the use of the laryngoscope. This is a golden rule which ought never to be forgotten.

At a meeting of the British Laryngological and Rhinological Association, held on March 25th, 1892, Dr. Sandford[1] opened a very important discussion on the necessity of systematic voice-training in public speakers. He brought forward the extremely common, and therefore interesting, case of a clergyman endowed by Nature with a vigorous, powerful vocal organ, but who, through faulty use, had got it into such a state of hyperæmia and debility as to imperil his carrying on his vocation. All of us who are examining throat cases constantly meet with just such patients every day. We can cure them as long as they rest their voices; but unless we insist on their putting themselves under a voice trainer they are certain to return to us as bad as ever after a few months of voice use.

Dr. Davies showed his little boy, who had been stammering a good deal after whooping cough, and who was cured by removal of adenoids, shortening the uvula, and a course of voice training of three months' duration. Dr. Matheson referred especially to stammering, and very rightly laid much stress on the necessity for accurate examination and treatment of the nose and naso-pharynx, abnormal condition of which probably accounts for 50 per cent. of all cases. Mr. Behnke related particulars of a case, showing that it is useless to attempt to cure stammering unless large tonsils, adenoids, and such abnormalities are first of all removed by the surgeon. Then he trains the action of the diaphragm by a suitable "diaphragmatic drill." Lastly, the confidence—nay, the affection—of the pupil is to be gained. Mr. Lennox Browne insisted on the importance of properly carried out diaphragmatic action, as opposed to any muscular effort which raises the clavicles, in order that the voice should stand wear and tear. He pointed out that the French method of voice production led to the unfortunate fault known as "vibrato," or "tremolo," and that the voice more quickly fell to pieces from the fact that the diaphragm was inefficiently trained.

Altogether the discussion was most valuable, as all who are consulted by voice users will readily admit.

BARCLAY J. BARON.

[1] *Journ. Laryn., Rhin., & Otol.*, May, 1892, p. 215.

